# In-Depth Insight into the Effect of Hydrophilic-Hydrophobic Group Designing in Amidinium Salts for Perovskite Precursor Solution on Their Photovoltaic Performance

**DOI:** 10.3390/nano12213881

**Published:** 2022-11-03

**Authors:** Guohua Wu, Hua Li, Shuai Chen, Shengzhong (Frank) Liu, Yaohong Zhang, Dapeng Wang

**Affiliations:** 1 Qingdao Innovation and Development Base of Harbin Engineering University, Harbin Engineering University, Harbin 150001, China; 2 Key Laboratory of Applied Surface and Colloid Chemistry, National Ministry of Education, Shaanxi Key Laboratory for Advanced Energy Devices, Shaanxi Engineering Laboratory for Advanced Energy Technology, School of Materials Science and Engineering, Shaanxi Normal University, Xi’an 710119, China; 3 Department of Engineering Science, Faculty of Informatics and Engineering, The University of Electro-Communications, Chofu, Tokyo 182-8585, Japan; 4 School of Physics, Northwest University, Xi’an 710127, China; 5 Shaanxi Key Laboratory for Carbon Neutral Technology, Xi’an 710127, China

**Keywords:** additive, amidinium salt, hydrophobic, high quality film, perovskite solar cells

## Abstract

Amidinium salts have been utilized in perovskite precursor solutions as additives to improve the quality of perovskite films. The design of hydrophilic or hydrophobic groups in amidinium salts is of great importance to photovoltaic device performance and stability in particular. Here we report a contrast study of a guanidinium iodide (GUI) additive with a hydrophilic NH_2_ group, and a N,1–diiodoformamidine (DIFA) additive with a hydrophobic C–I group, to investigate the group effect. The addition of GUI or DIFA was beneficial to achieve high quality perovskite film and superior photovoltaic device performance. Compared with GUI, the addition of the DIFA in a perovskite precursor solution enhanced the crystal quality, reduced the defect density, and protected the water penetration into perovskite film. The perovskite solar cell (PSC) devices showed the best power conversion efficiency (PCE) of 21.19% for those modified with DIFA, as compared to 18.85% for the control, and 20.85% for those modified with GUI. In benefit to the hydrophobic C–I group, the DIFA–modified perovskite films and PSC exhibited the best light stability, thermal stability, and humidity stability in comparison to the control films and GUI–modified films. Overall, the introduction of a hydrophobic group in the amidinium salts additive was demonstrated to be an efficient approach to achieve high quality and stable perovskite film and PSC devices.

## 1. Introduction

Since Kojima first reported organometal halide perovskites as visible-light sensitizers for photovoltaic cells and achieved a power conversion efficiency (PCE) of 3.8% in 2009 [1], organometal halide perovskite materials have been extensively employed in various photovoltaic applications, due to their outstanding opto-electric properties, including a high molar extinction coefficient and mobility, as well as long charge diffusion lengths and carrier lifetime [2]. So far, a certified PCE of 25.7% for perovskite solar cells (PSCs) have been achieved, which demonstrates their promising suitability for applications in the photovoltaic field [3]. Such remarkable progress for PSCs mainly profits from the development of a perovskite photoactive layer and that greatly stimulates its extensive research [4]. As we all know, the perovskite photoactive layer as a key part in PSC devices, and it plays crucial roles in harvesting light from the sun, as well as effectively separating and transferring the photogenerated charge carriers [5]. The high quality of the perovskite photoactive layer is of great importance to elevate the PSC photovoltaic performance, along with superior long–term device stability [6].

**Figure 1:**
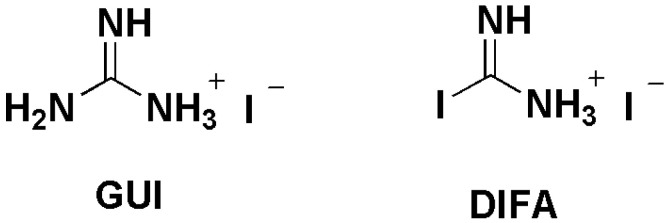
The molecular structures of GUI and DIFA.

Many effective passivation methods have been proposed to further reduce the grain boundaries, enlarge the perovskite grain size, or smooth the interface/surface of the perovskite film [7,8,9]. Additive engineering is one of the passivation methods that has been demonstrated as a highly effective strategy to obtain the high quality perovskite photoactive layer, mainly as a result of the coordination formation of additives with the under-coordinated Pb^2+^ or halide anions, which regulate the perovskite crystallization process, control the grain size or morphology of perovskite films, and therefore enhance the PSC photovoltaic performance upon the addition of an additive [10,11]. To date, lots of small additives for PSC devices, including inorganic additives [12,13,14] and organic additives [15,16], have been successfully utilized. In particular, ammonium salts [17] as typical additives, including hydrophobic ones, such as linear alkyl ammonium bromides/chloroform [18], 2–chloroethylamine and 2–bromoethylamine [19], caffeine [20], phenethylammonium iodide [21], and hydrophilic ones, such as 5–ammounium valeric acid iodide [22], guanidinum isothiocyanate [23], melaminium iodide [24], hydroxylamine hydrochloride [25], amidine [26], oleylammonium iodide, and phenethylammonium iodide [27], have been widely utilized as additives for highly efficient and stable PSCs. These research implicate that the functional group properties of molecular passivation additives that result in different hydrophilic or hydrophobic properties play a pivotal role in the formation of high quality perovskite photoactive layers. However, scarce research has been conducted with a comprehensive study of the effect of hydrophobic or hydrophilic groups in ammonium salts on photovoltaic performance to understand the design rules for the passivation additive molecules. In 2016, the additive guanidinium iodide (GUI) had been demonstrated to successfully suppress the trap density of MAPbI_3_ film without guanidinium ions incorporating in the perovskite lattice [28]. Later, we synthesized a novel organic additive named DIFA with a similar structure to GUI, which was created from C–I instead of the C–NH_2_ in GUI, as shown in Figure 1. These two molecules give us an opportunity to reveal the effect of hydrophobic or hydrophilic groups on the performance of amidinium salt–based, highly efficient PSCs. Thus, in this study, identical FA_0.85_MA_0.15_PbI_3_ (FA = HC(NH_2_)_2_, MA = CH_3_NH_3_) PSC devices with GUI or DIFA additives were studied in detail, which provided a theoretical guide for developing novel additives for PSCs.

## 2. Materials and Methods

Materials used in the experiments included MAI, FAI, PbI_2_, Spiro–OMeTAD, and GUI, which were purchased from the Xi’an Polymer Light Technology Corp. (Xi’an, China). The DIFA was synthesized using the method reported by the previous literature [29].

### 2.1. Device Fabrication

Fluorine–doped tinoxide (FTO) glass (2.5 cm × 2.5 cm) was first cleaned and then treated with O_3_ plasma for 15 min. Then, the FTO substrate was immersed in a 40 mM TiCl_4_ aqueous solution at 70 °C for 1 h to prepare the TiO_2_ layer (~40 nm). The above FTO/TiO_2_ substrate was annealed at 200 °C for 30 min in air. The control perovskite film was deposited using a DMF/DMSO (1 mL, v/v 4:1) mixture precursor solution of FAI, MAI, and PbI_2_, with a molar ratio of 0.85:0.15:1. Subsequently, molar ratios of 2% DIFA or 1% GUI additive with respect to the PbI_2_ in the pristine perovskite precursor solution were added to prepare the DIFA or GUI modified perovskite film, or the target solar cells. Then, the spin–coating technique was utilized for the deposition process of the precursor solutions, under inert atmosphere inside a nitrogen glovebox, with a small amount chlorobenzene employed as an anti–solvent. Then, the control perovskite film was placed on a hot plate at 150 °C for 30 min. Different amounts of GUI or DIFA in the control precursor solution were added to deposit the GUI or DIFA modified perovskite films, using the spin–coated and annealed process with the same procedure as for the control perovskite film. Subsequently, the spiro–OMeTAD layer was deposited on top of the above perovskite films by spin–coating. At last, gold electrodes with thicknesses of ca. 100 nm were thermally evaporated on top of the spiro–OMeTAD layer. The device area for the fabricated solar cells was 0.09 cm^2^.

### 2.2. Instruments and Characterization

A field emission scanning electron microscope (SEM, SU–8020, JEOL, Tokyo, Japan) was used to measure the surface morphology images of the control, GUI–, and DIFA–modified perovskite films. Atomic force microscopy (AFM) images were performed with a Bruker Dimension ICON Scanning Station by using conducting AFM tips (SCMPIT/PtIr, Camarillo, CA, USA). A contact angle of a tiny water droplet on the perovskite film was measured on an OCA20 instrument to investigate the hydrophilic or hydrophobic properties (Dataphysics, Filderstadt, Germany). A UV−vis spectrophotometer (UV-3600, Shimadzu, Kyoto, Japan) was utilized to measure the absorption behavior of the control, GUI–, and DIFA–modified perovskite films. The photoluminescence (PL) spectra with an excitation at 510 nm were measured using a FLS980 spectrometer (Edinburgh Instruments Ltd., Livingston, UK). The time–resolved photoluminescence (TRPL) spectra were measured using FluoQuant 300 spectrometer (PicoQuant, Berlin, Germany). X-ray diffraction (XRD) patterns of the fresh or aged perovskite films were performed on a Bruker D8 X-ray Diffractometer instrument with a GADDS detector (Karlsruhe, Germany). The *J–V* curves of the control, GUI–, and DIFA–modified PSC devices were detected by a Keithley Model 2400 digital source meter with an illumination intensity of 100 mW cm^−2^ (AM 1.5G, SAN–EI, Enlitech, Shanghai, China). The corresponding external quantum efficiency (EQE) spectra were measured on a QTest Station 2000ADI system (Crowntech. Inc., Macungie, PA, USA) with a silicon reference. Electrochemical impedance spectroscopy (EIS) of the PSC devices was measured using a bias value equal to the voltage under dark conditions, with a frequency range from 10 Hz to 4 × 10^6^ Hz (Zennium, Zahner, Kronach, Germany).

## 3. Results

**Figure 2:**
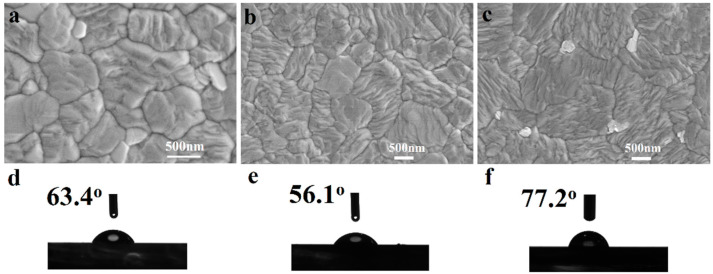
Scanning electron microscope (SEM) images (**a**–**c**) and water contact angles (**d**–**f**) of the pristine film (**a**,**d**), GUI–modified perovskite film (**b**,**e**), and DIFA–modified perovskite film (**c**,**f**).

SEM images of the pristine perovskite film and GUI– or DIFA–modified perovskite films are illustrated in Figure 2a–c. The average grain size of the pristine perovskite film was about 500 nm. Larger grain sizes have been observed for GUI– or DIFA–modified perovskite films. Clearly, the addition of GUI or DIFA can significantly increase the perovskite crystal grain size, compared with the pristine perovskite film, which indicates that the addition of GUI or DIFA slow down the growth rate of the perovskite material, and thus effectuate high quality perovskite films. As seen in the SEM cross–section images as shown in Figure S1, the thickness of the perovskite active layer was about 410 nm. The contact angles of tiny water droplets on the perovskite film are shown in Figure 2d–f. The water contact angle of the DIFA–modified perovskite film was the largest (77.2°), followed by the pristine perovskite film (63.4°), and the GUI–modified perovskite film (56.1°). This resulted from the hydrophobic nature of the C–I in the DIFA and hydrophilic nature of the C–NH_2_ in the GUI. Therefore, the largest water contact angle of the DIFA–modified perovskite film would be beneficial to prevent water infiltration and ultimately enhance the PSC humidity stability, while the GUI–modified perovskite film would exhibit the opposite effect. Figure S2 shows the AFM height images of the control, GUI– and DIFA–modified perovskite films on the TiO_2_ layer. The root–mean–square (RMS) roughness was 21.0 nm for the control perovskite film, 18.5 nm for the GUI–modified perovskite film, and 18.2 nm for the DIFA–modified perovskite film, respectively. The smallest RMS value for the DIFA–modified perovskite film indicated that the DIFA could facilitate the flatness of the perovskite films, perhaps induced by the strong interaction between the perovskite film and the DIFA molecules.

**Figure 3:**
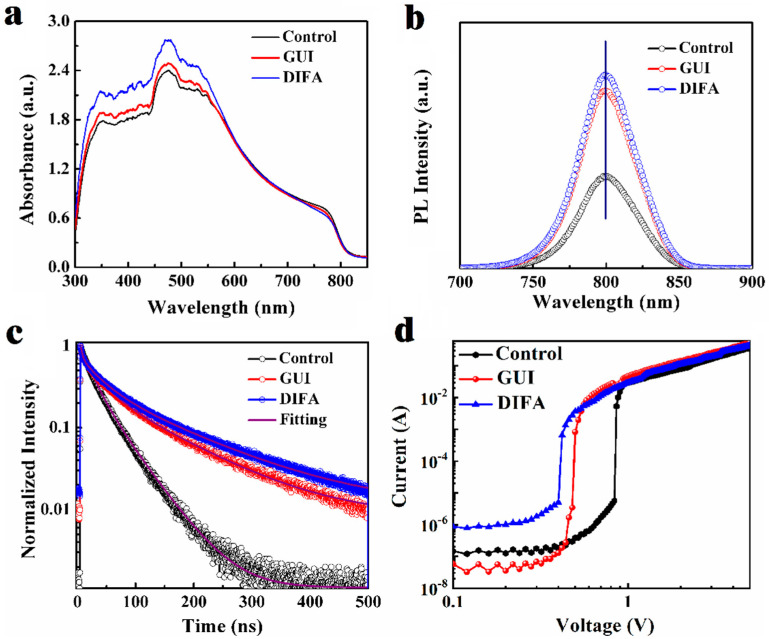
Absorption spectra (**a**), steady-state photoluminescence spectra (**b**), transient-state photoluminescence spectra (**c**), and dark current–voltage characteristics of electron only devices (FTO/TiO_2_/perovskite/PCBM/Ag) (**d**) of pristine perovskite films, GUI– and DIFA–modified perovskite films.

In order to obtain the effect of the GUI and DIFA additives on the perovskite photoactive layer, the UV–vis absorption spectra were conducted, as shown in Figure 3a. The absorption intensity was improved by the introduction of the GUI and DIFA. The stronger absorption intensity for the DIFA–modified perovskite film, which was more than the GUI–modified film or the control film, was more relevant to its larger perovskite grains, which therefore enhanced the short circuit current (*J*_sc_) of the PSCs. The calculated bandgaps from the Tauc plots of control, GUI–, and DIFA–modified perovskite films, as shown in Figure S3, were about 1.540, 1.541, and 1.538 eV, respectively. Due to these values being so close, the bandgaps of the three samples were approximately the same, at 1.54 eV. The steady–state photoluminescence (PL) curve, as shown in Figure 3b, and normalized time–resolved photoluminescence (TRPL) curve, as shown in Figure 3c, were then obtained to explore the charge dynamics. For the pristine perovskite films, the PL intensity of the GUI–modified and DIFA–modified perovskite films increased progressively with almost no PL peak shift, which confirmed the effective defect passivation of the GUI and DIFA with the gradually reduced trap–assisted recombination. After a bi-exponential decay fitting for the TRPL spectra, a fast component (*τ*_1_) and a slow component (*τ*_2_) were concluded in Table S1. The longest carrier lifetime was obtained for the DIFA–modified perovskite film, followed by the GUI–modified film, and the pristine film. This indicated that the addition of GUI and DIFA helped to suppress the interface charge recombination of the device. The space–charge–limited current (SCLC) measurement was then performed to quantify the density of trap states in the pristine, GUI–modified, or DIFA–modified perovskite films. The corresponding *J–V* curves of the FTO/TiO_2_/perovskite (pristine, GUI–modified, or DIFA–modified)/PCBM/Ag electron devices under dark conditions are illustrated in Figure 3d. The trap–filled limit voltages could be obtained from the curves as 0.91 V for the control perovskite film, 0.44 V for the GUI–modified perovskite film, and 0.40 V for the DIFA–modified perovskite film. According to the trap–state density (*n*_trap_) equation [29], the accurate trap densities were 2.30 × 10^16^ cm^−3^ for the control perovskite film, 1.11 × 10^16^ cm^−3^ for the GUI–modified perovskite film, and 1.02 × 10^16^ cm^−3^ for the DIFA–modified perovskite film. It can be seen that the trap density trend was consistent with the PL and TRPL results. The result qualitatively indicated that the trap density for the control perovskite film was larger than the GUI– and DIFA–modified perovskite films. The more traps there are, the larger the charge nonradiative recombination, which leads to open circuit voltage (*V*_oc_) lost and current leakage, which affects the fill factor (*FF*) value for solar cell devices. The introduction of GUI or DIFA in the perovskite precursor solution not only stimulated the growth of perovskite crystals, but also effectively diminished the Pb–I anti–site defect densities in the perovskite film, which were induced by undercoordinated Pb atoms [30].

**Figure 4:**
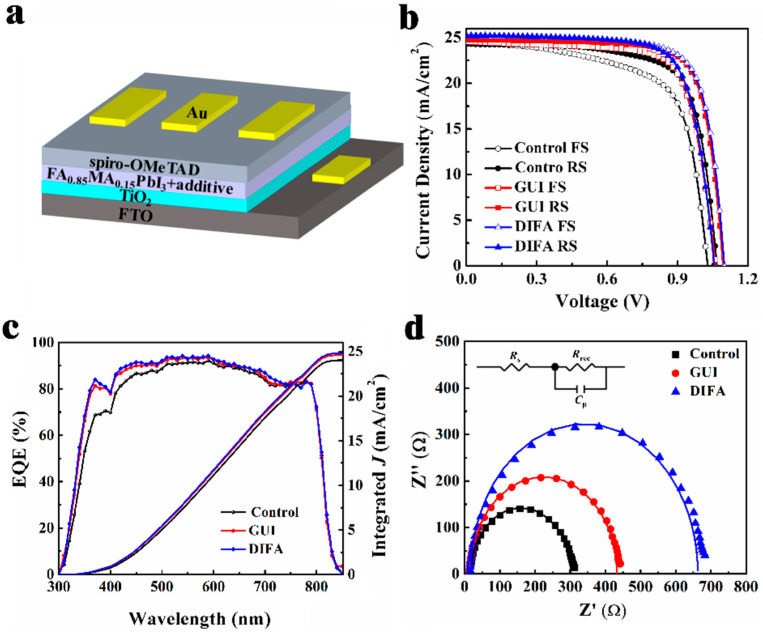
Schematic of the device architecture (**a**), current density–voltage (*J–V*) under different scans (**b**), external quantum efficiency (EQE) spectra (**c**), and Nyquist plots under dark conditions (**d**) of pristine perovskite films, GUI– and DIFA–modified perovskite films.

The PSC structure is illustrated in Figure 4a. Here, the additives were GUI and DIFA, respectively. As shown in Figure 4b, the *J*–*V* curves for the control, GUI–, and DIFA–modified PSC devices are presented. The corresponding average key photovoltaic parameters of 50 individual PSC devices are summarized in Table 1 and the statistical distribution for these PSCs is shown in Figure S4. The control PSC device, made of pristine perovskite film without additives, showed the best PCE of 18.85%, with a *J*_sc_ of 24.21 mA·cm^−2^, a *V*_oc_ of 1.08 V, and a *FF* of 71.8%, which are concluded in Table S2. Upon the addition of the GUI, the best PSC device showed a much better photovoltaic performance, with a PCE of 20.85%, along with an increased *V*_oc_, *J*_sc_, and FF of 1.10 V, 24.64 mA·cm^−2^, and 77.1%, respectively. Meanwhile after the addition of DIFA, the best PCE sharply increased to 21.19% with an impressive improvement of >10% of the control cell, and yielded an increased *J*_sc_, *V*_oc_, and FF of 25.04 mA·cm^−2^, 1.10 V, and 77.2%, respectively. It is worth noting that both the addition of GUI and DIFA could substantially enhance the photovoltaic performance, especially the *J*_sc_ and FF values. The DIFA–modified PSC exhibited a better PCE because of the enhancement of the *J*_sc_ value, which resulted from its higher crystallinity of perovskite film upon the addition of DIFA, when compared to the GUI–modified film, as suggested by the XRD results. Hysteresis phenomenon were also further investigated by comparing the reverse (RS) and forward (FS) *J–V* photovoltaic performance. Hysteresis indices were obtained as differential values between the PCE in reverse scan and PCE in forward scan, divided by the PCE in forward scan. The reverse (RS) and forward (FS) *J–V* curves of the control, GUI–, and DIFA–modified PSC devices are shown in Figure 4b, and the corresponding hysteresis indices are summarized in Table S3. Hysteresis indices of 12.4 for the control device, 7.6 for the GUI–modified PSC, and 5.1 for the DIFA–modified PSC were obtained. The addition of GUI and DIFA in particular could effectively diminish the hysteresis phenomenon in the PSCs, due to their reduced defects in the perovskite films.

Then we further verified the conformity relationship between the tested *J*_sc_ values (24.21 mA·cm^−2^ for the control PSC device, 24.64 mA·cm^−2^ for the GUI–modified PSC device, and 25.04 mA·cm^−2^ for the DIFA–modified PSC device) from the *J–V* curves, and calculated the *J*_sc_ values (24.02 mA·cm^−2^ for the control PSC device, 24.62 mA·cm^−2^ for the GUI–modified PSC device, and 24.82 mA·cm^−2^ for the DIFA–modified PSC device) that were integrated over the external quantum efficiency (EQE) spectra, as shown in Figure 4c. To investigate the charge transport process, the Nyquist plots of the GUI– or DIFA–modified PSC devices in comparison with the control obtained from electrochemical impedance spectra (EIS), using a 1.05 V bias under dark conditions, are shown in Figure 4d. Fitting values for the Nyquist plots of the control, GUI–, and DIFA–modified PSC devices are collected in Table 2. As displayed in the inset of Figure 4a, a simple equivalent circuit diagram with a series resistance (*R*_s_) and a recombination resistance (*R*_rec_), in parallel with a chemical capacitance (*C*_μ_) at the TiO_2_/perovskite/HTL interface, was employed. The *R*_s_ values were 17.6 Ω for the control PSC, 15.4 Ω for the GUI–modified PSC, and 13.2 Ω for the DIFA–modified PSC. This trend was opposite from the order of *FF*s of the respective devices, which indicated that the addition of GUI or DIFA could reduce the resistance of the perovskite film by the passivation effect. Higher respective *R*_rec_ values of 418 Ω and 646 Ω for the GUI– and DIFA–modified PSC devices were obtained, compared with 284 Ω for the control device, which indicated that the addition of GUI or DIFA could abate the recombination rate at the TiO_2_/perovskite/HTL interface with more effective suppression of the charge recombination, resulting in better *V*_oc_ values. By comparison, the DIFA–modified PSC exhibited the lowest *R*_s_ value and the highest *R*_rec_ value, and revealed the best interfacial contact with suppressed interfacial recombination, which was favorable for the enhancement of the *FF* and *V*_oc_ values [31].

**Figure 5:**
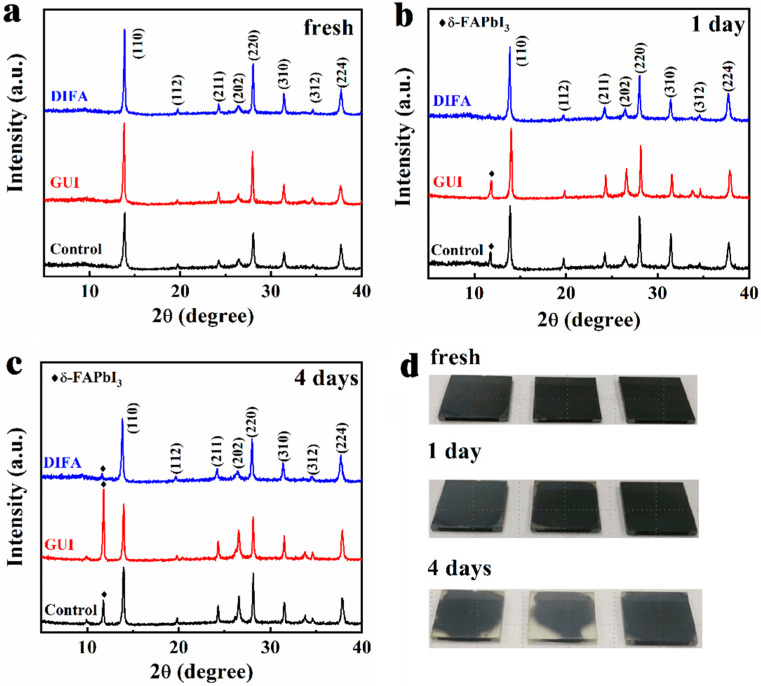
XRD patterns of the control, GUI–, and DIFA–modified perovskite films with a symbol ♦ denoting the diffraction peak of the *δ*–phase FA–based perovskite: (**a**) fresh, (**b**) after one day, and (**c**) after four days, (**d**) The photographs of the control, GUI–, and DIFA–modified perovskite films aged for different amount of time under a high humidity of ~60% condition.

X-ray diffraction (XRD) patterns of thin pristine perovskite film, GUI–, and DIFA–modified perovskite films are illustrated in Figure 5a. Compared with the pristine perovskite film, the XRD diffraction intensities at 13.8° and 28.0° originating from the (110) and (220) planes of the 3D perovskite related features were enhanced upon the addition of GUI and DIFA, which indicated improved perovskite crystallinity for the GUI– and DIFA–modified perovskite films. In comparison with the XRD pattern of the control perovskite film, the XRD of the GUI–modified perovskite film showed almost no change. The van der Waals radii of the NH_2_ group was about 1.46 Å, which was shorter than that of the I group (2.20 Å) [32]. Therefore, the DIFA molecule would not go into the lattice of perovskite, which was agreed with the XRD result. The pictures of the fresh films, after one day, and after four days are presented in Figure 5a–c. Obviously, the GUI–modified perovskite films went from black to grey or colorless. There was no *δ*–FAPbI_3_ phase in the fresh control, GUI–, or DIFA–modified perovskite films. After one day, the *δ*–FAPbI_3_ phase appeared for the control and the GUI–modified perovskite films. The control films and the GUI–modified perovskite films generated more and more reinforced *δ*–FAPbI_3_ phase intensity, as illustrated in Figure 5, after being stored in ~55% RH atmosphere for 4 days. It was surprising that a very weak *δ*–FAPbI_3_ phase intensity was shown for the DIFA–modified perovskite film, which basically resulted from the hydrophobic C–I bond in the DIFA molecule. The phase transition of the FA–based perovskite film easily generated from the dark cubic α–phase to the yellow orthorhombic *δ*–phase under ambient conditions [33]. The FA–based perovskite photovoltaic performance mainly depended on the dark cubic α–phase content in the FA–based perovskite film. The photovoltaic performance degradation generally resulted from the light harvest and charge transport efficiency decreases induced by the photoinactive *δ*–phase of the FA–based perovskite [34]. Thus, it was strongly essential to stabilize the α–phase of the FA–based perovskite, or suppress the unwanted δ–phase commonly formed by proper means. The addition of DIFA in the FA–based perovskite precursor solution could effectively inhibit the phase transition of the FA–based perovskite from the α–phase to the δ–phase, as suggested by the above X-ray diffraction (XRD) measurements.

**Figure 6:**
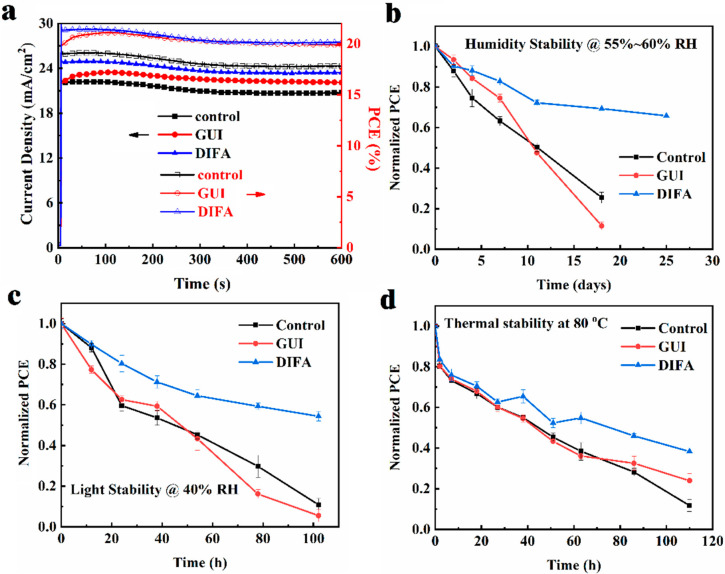
Stable output *J*_max_ and PCE curves (**a**), environmental stability of the PSCs with a relative humidity of 55~60% (**b**), light stability of PSCs in xenon lamp aging box with a relative humidity of ≈40% (**c**), thermal stability of PSCs at 80 °C under N_2_ atmosphere (**d**).

The stabilized photocurrent density and PCE output curves for the control, GUI–, and DIFA–modified PSC devices under light soaking at under maximum power point (MPP) conditions were investigated, as shown in Figure 6. After about 600 s of light soaking, the PCEs degraded to 17.88%, 19.89%, and 20.19% for the control, GUI–, and DIFA–modified PSCs, respectively. The change trend for photocurrent density was the same as that of PCE. By contrast, the GUI–modified PSC device revealed a less stable behavior over time. This indicated that the addition of DIFA in the perovskite film was more beneficial to ameliorate the device light soaking stability than for the GUI. The air stability of the unencapsulated PSC devices was measured with a relative humidity of 55–60% in a humidity chamber for 25 days, and the results are shown in Figure 6b. The DIFA–modified PSC device exhibited a 30% degradation in its initial efficiency after 18 days, while the control and GUI–modified ones degraded to 25% and 11%, respectively. The light stability of the unencapsulated control, GUI–, and DIFA–modified PSC devices under one sun illumination is shown in Figure 6c. The DIFA–modified PSC device maintained ~55% of its initial efficiency after 110 h, whereas the control and GUI PSC devices rapidly degraded to 11% and 5% of their initial efficiencies, respectively, during the same period, indicating that the addition of DIFA could enhance the light stability compared with the GUI. The significant PCE degradation under continuous light soaking for the control, GUI–, and DIFA–modified PSC devices was probably caused by the deteriorated perovskite active layer under the high humidity environment, as demonstrated by XRD results. The thermal stability of the unencapsulated PSC devices was measured in N_2_ atmosphere at a continuous 80 °C is shown in Figure 6d. The DIFA–modified PSC device degraded by 62% during 110 h, while the control and GUI–modified device degraded to 12% and 24% of their initial efficiencies, respectively. These results indicate that the DIFA–modified PSC device exhibited significantly enhanced air stability and light stability compared with the control and GUI–modified PSC devices, which could arise from the enhanced phase stability of the DIFA–modified perovskite film with relatively stronger hydrophobicity [35].

## 4. Conclusions

Additive engineering has been demonstrated as an efficient means to improve the photovoltaic performance of PSCs. In this study, GUI with a hydrophilic NH_2_ group and DIFA with a hydrophobic C–I group were utilized as additives in perovskite precursor solutions to investigate the effect of hydrophilic or hydrophobic group designing in amidinium salts on their photovoltaic performance. Compared with the control perovskite film or introducing GUI in particular, higher quality perovskite film was obtained upon the addition of DIFA with less trap density, larger grain size, and relatively hydrophobic properties. Therefore, DIFA–modified PSC exhibited an outstanding PCE of 21.19%, which was 12.41% and 1.63% higher than the control and GUI–modified PSC. After continuous light, thermal, and humidity treatments, the DIFA–modified PSC exhibited outstanding stability due to its hydrophobic C–I group. Through comparison, it was found that hydrophobic groups in amidinium salt additives could relatively enhance the photovoltaic performance of perovskite solar cells. More importantly, hydrophobic groups in amidinium salt additives play vital roles in designing stable perovskite solar cells.

## Figures and Tables

**Table 1:** Average photovoltaic parameters of the control, GUI, and DIFA modified PSC devices under reverse scans.

PSCs	*V*_oc_/V	*J*_sc_/mA·cm^−2^	*FF*/%	PCE/%
Control	1.06 ± 0.02	24.39 ± 0.33	71.1 ± 1.7	18.41 ± 0.25
GUI	1.09 ± 0.01	24.76 ± 0.17	75.2 ± 1.4	20.22 ± 0.33
DIFA	1.09 ± 0.01	24.72 ± 0.41	76.3 ± 0.9	20.65 ± 0.37

**Table 2:** Fitting values for the Nyquist plots of the control, GUI–, and DIFA–modified PSC devices under 1.05 V bias.

PSCs	*R*_s_/Ω	*R*_rec_/Ω	*C*_μ_/F
Control	17.6	284	1.13 × 10^−8^
GUI	15.4	418	9.69 × 10^−9^
DIFA	13.2	646	1.11 × 10^−8^

## Data Availability

The data that support the findings of this study are available from the corresponding author upon reasonable request.

## Supplementary Materials

The following supporting information can be downloaded at: https://www.mdpi.com/article/10.3390/nano12213881/s1, Figure S1: Cross–sectional SEM images of PSC structure (a) the control, (b) the GUI–modified PSC, and (c) the DIFA–modified PSC. Figure S2: AFM (atomic force microscopy) height images of the pristine film, GUI–modified perovskite film, and DIFA–modified perovskite film; Figure S3: The calculated bandgaps from Tauc plots of control, GUI– and DIFA–modified perovskite films; Figure S4: Statistical distribution for a batch of 50 perovskite solar cells for the control PSCs, GUI–modified PSCs, and DIFA–modified PSCs; Table S1: Fitting parameters of time-resolved PL spectra based on the control, GUI–, and DIFA–modified perovskite films; Table S2: The best photovoltaic parameters of the control, GUI–, and DIFA–modified PSC devices under reverse scans, Table S3: Summary of photovoltaic parameters of the control, GUI–, and DIFA–modified PSC devices under reverse scan and forward scan and the corresponding hysteresis indices.
