# Supplementary material for: In-Depth Insight into the Effect of Hydrophilic-Hydrophobic Group Designing in Amidinium Salts for Perovskite Precursor Solution on Their Photovoltaic Performance
Source: Nanomaterials (Basel). 2022 Nov 3;12(21):3881. doi: 10.3390/nano12213881 (PMC9656357; doi:10.3390/nano12213881)
Supplement: Supplementary file 1 [file nanomaterials-12-03881-s001.zip › nanomaterials-1961075-supplementary.pdf]

nanomaterials

In-Depth Insight into the Effect of Hydrophilic-Hydrophobic Group Designing in Amidinium Salts for Perovskite Precursor Solution on Their Photovoltaic Performance

Guohua Wu [1,2], Hua Li [3], Shuai Chen [2], Shengzhong (Frank) Liu [2], Yaohong Zhang [4,5,*] and Dapeng Wang [2,*]

[1] Qingdao Innovation and Development Base of Harbin Engineering University, Harbin Engineering University, Harbin 150001, China
[2] Key Laboratory of Applied Surface and Colloid Chemistry, National Ministry of Education, Shaanxi Key Laboratory for Advanced Energy Devices, Shaanxi Engineering Laboratory for Advanced Energy Technology, School of Materials Science and Engineering, Shaanxi Normal University, Xi'an 710119, China
[3] Department of Engineering Science, Faculty of Informatics and Engineering, The University of Electro-Communications, Chofu, Tokyo 182-8585, Japan
[4] School of Physics, Northwest University, Xi'an 710127, China
[5] Shaanxi Key Laboratory for Carbon Neutral Technology, Xi'an 710127, China
* Correspondence: yhzhang@nwu.edu.cn (Y.Z.); dpwang@snnu.edu.cn (D.W.)

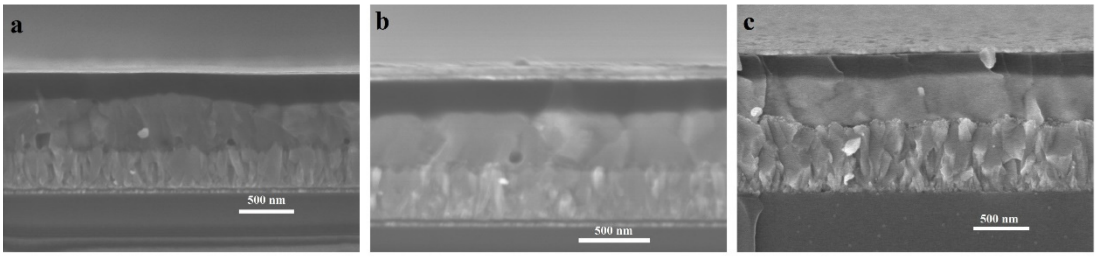

Figure S1. Cross-sectional SEM images of PSC structure (a) the control one, (b) the GUI modified one, and (c) the DIFA modified one.

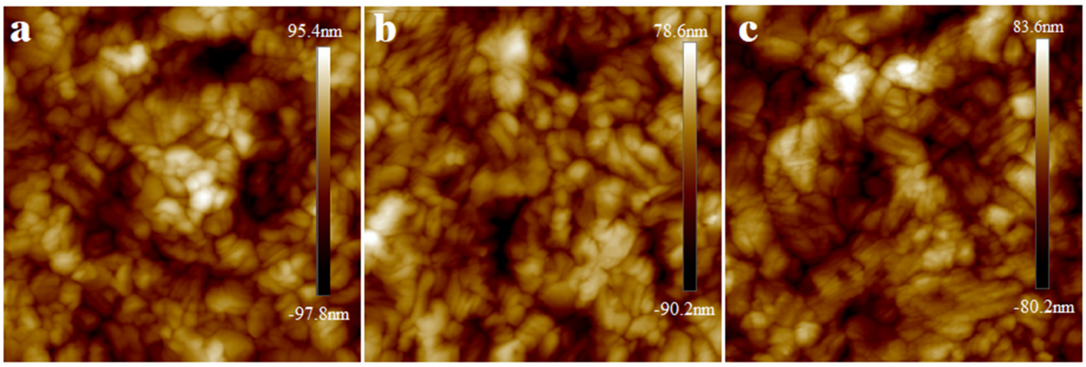

Figure S2. AFM (atomic force microscopy) height images of the pristine film (a), GUI modified perovskite film (b), and DIFA modified perovskite film (c).

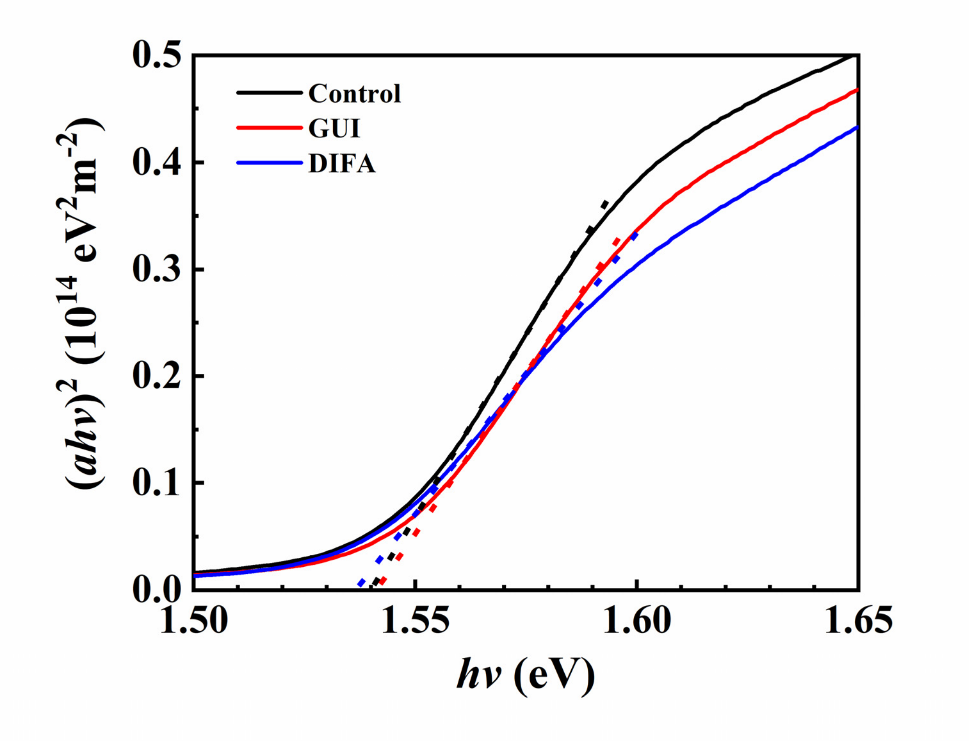

Figure S3. The calculated bandgaps from Tauc plots of control, GUI and DIFA modified perovskite films.

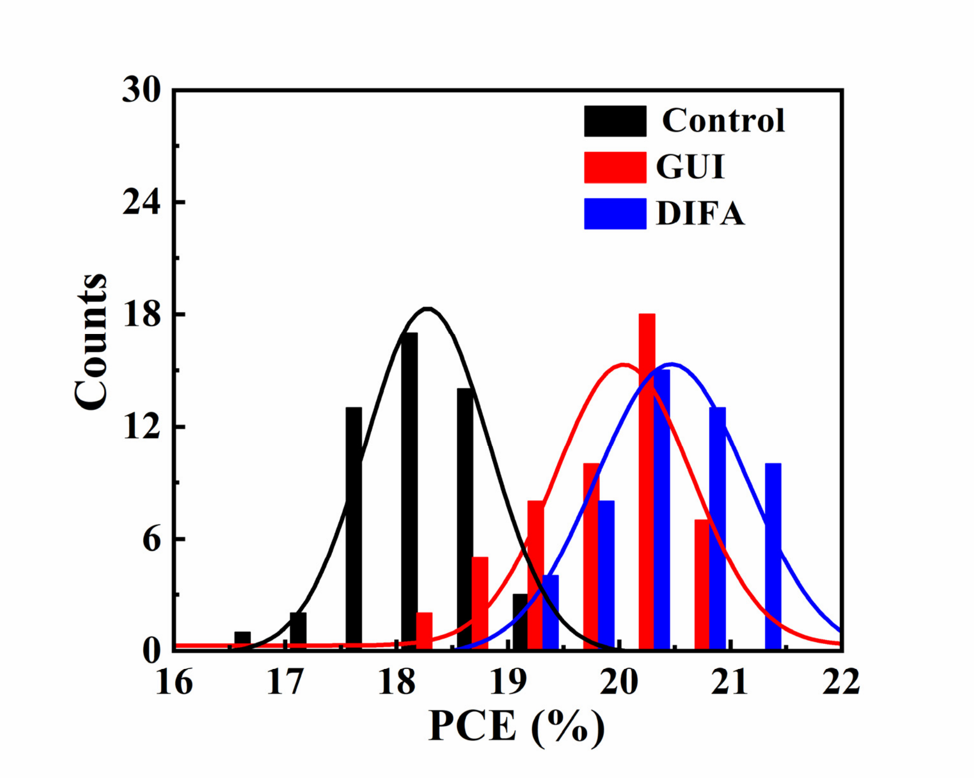

Figure S4. Statistical distribution for a batch of 50 perovskite solar cells for the control ones, GUI modified ones, and DIFA modified ones.

Table S1. Fitting parameters of time-resolved PL spectra based on the control, GUI, and DIFA modified perovskite films.

Sample	τ_1(ns)	Amplitude A_1(%)	τ_2(ns)	Amplitude A_2(%)	τ_{ave}(ns)
Control	44.07	43.50	17.61	56.48	35.03
GUI	107.37	42.40	24.30	57.60	87.83
DIFA	135.09	42.31	26.70	57.69	112.08

Table S2. The best photovoltaic parameters of the control, GUI, and DIFA modified PSC devices under reverse scans.

PSCs	V_{oc} (V)	J_{sc} (mA·cm^{-2})	*FF* (%)	PCE (%)
Control	1.08	24.21	71.8	18.85
GUI	1.10	24.64	77.1	20.85
DIFA	1.10	25.04	77.2	21.19

Table S3. Summary of photovoltaic parameters of the control, GUI, and DIFA modified PSC devices under reverse scan and forward scan and the corresponding hysteresis index.

		V_{oc} (V)	J_{sc} (mA·cm^{-2})	FF (%)	PCE (%)	Hysteresis Index (%)
Control	Reverse	1.05	24.45	71.9	18.45	12.4
	Forward	1.01	24.32	66.9	16.41	
GUI	Reverse	1.09	24.77	76.5	20.73	7.6
	Forward	1.06	24.55	73.3	19.15	
DIFA	Reverse	1.08	25.26	76.4	20.80	5.1
	Forward	1.06	25.26	74.2	19.80	
